# Preventing microbial biofilms on catheter tubes using ultrasonic guided waves

**DOI:** 10.1038/s41598-017-00705-8

**Published:** 2017-04-04

**Authors:** Huanlei Wang, Fengmeng Teng, Xin Yang, Xiasheng Guo, Juan Tu, Chunbing Zhang, Dong Zhang

**Affiliations:** 1 Key Laboratory of Modern Acoustics (MOE), Department of Physics, Collaborative Innovation Centre of Advanced Microstructure, Nanjing University, Nanjing, 210093 China; 2 Department of Applied Engineering, Zhejiang Business College, Hangzhou, 310053 China; 3 Department of Laboratory Medicine, TCM of Jiangsu Province, Nanjing, 210012 China; 4 The State Key Laboratory of Acoustics, Chinese Academy of Science, Beijing, 10080 China

## Abstract

Biofilms on indwelling tubes and medical prosthetic devices are among the leading causes of antibiotic-resistant bacterial infections. In this work, a new anti-biofilm catheter prototype was proposed. By combining an endotracheal tube (ET) with a group of ultrasonic guided wave (UGW) transducers, the general idea was to prevent bacteria aggregation with UGW vibrations. Based on quantitative analysis of UGW propagation, detailed approach was achieved through (a) selection of ultrasonic frequency, wave modes and vibration amplitude; and (b) adoption of wave coupling and 45° wave incidence technique. Performance of the proposed UGW-ET prototype was demonstrated via *in vitro* experiments, during which it deterred deposition of *Pseudomonas aeruginosa* (*P. aeruginosa*) biofilms successfully. With current configuration, UGW amplitudes ranged from 0.05–5 nm could be optimal to achieve biofilm prevention. This work sheds a light in the underlying mechanism of ultrasound-mediated biofilm prevention, and will inspire the development of new catheters of better antibacterial capability.

## Introduction

Indwelling devices are widely used in clinical treatments, like catheter lines, prosthetic devices, contact lenses, heart valve replacements, and other surgical implants, *etc*. Unfortunately, these devices are also breeding grounds for microbial biofilms, which usually cause bacterial infections. In the development of ventilator-associated pneumonia, one of the culprits is the biofilm on ETs^1, 2^, most commonly caused by aggregation of *P. aeruginosa* ^3^. For patients with cystic fibrosis or chronic bronchiectasis, *P. aeruginosa* biofilms on catheters are also blamed for chronic infections^4, 5^. According to published reports, over 80% microbial infections in human bodies were contributed by biofilms, among which 65% cases were related to those on indwelling devices^6–8^. As a consequence, the mortality rate of patients undergoing long-term catheterization was significantly increased^9^.

Once established, biofilms could build up a protective growth mode, helping bacteria to resist antibiotics and immune systems^9–14^. For patients undergoing surgeries, antibiotics then cost over one-third of their expenses on average^15^. Abuse of antibiotics helps build up the vicious cycle of antibiotics resistance, which in turn makes treatments more difficult^16^. Many efforts have been made to develop chemical and mechanical approaches for biofilm prevention. Catheters coated with specified antimicrobial materials, including hydrogel, silver salts, silver nanoparticles and antimicrobials, *etc*., have been proven effective^17–20^. Nevertheless, biofilms could still emerge, especially on catheters that remain inside bodies for relatively long periods, e.g., urinary catheters and endotracheal tubes. What makes the situation even worse is, once bacteria start to aggregate on tube surfaces, the antimicrobial effect of above-mentioned approaches might be significantly reduced^21^. Therefore, the problem of biofilm prevention is far from been solved, and there is an urgent demand in developing more effective approaches.

Acoustic wave, benefiting from its vibrational nature, possesses the potential in deterring biofilm deposition. Previous studies show that ultrasound could help to decrease antibiotics-resistance of biofilms^9, 10, 22–25^. According to Carmen *et al*., combination of ultrasound and antibiotics could reduce the generation of Escherichia coli biofilms^26^. In their experiments 28.5-kHz ultrasound was used, working at an intensity of 500 mW/cm^2^ and a duty cycle of 33.3%. Hazan *et al*. chose a 100–300 kHz ultrasonic transducer operating at 300–800 nm amplitude, and spread surface acoustic waves (SAWs) into catheters, which in turn helped to diminish the development of *Escherichia coli* biofilm^9^. Kopel et al. then reported that, 100-kHz SAWs causing 0.2–2 nm catheter vibration could achieve the same goal as well^10^. It is also interesting that, 95–220 kHz SAWs of 0.4-W/cm^2^ intensity could enhance neutrophil killing of *Staphylococcus epidermidis* ^23^. Thus, ascribed to its advantages of being non-invasive, non-chemical, inexpensive and portable, ultrasound becomes very attractive and promising in developing antibacterial catheters. However, up to date, the underlying mechanism through which ultrasonic waves could prevent biofilm formation is still unclear. It is here expected that, with full analysis/regulation of wave motions in catheters, capability of ultrasonic waves on biofilm prevention could be highly elevated.

The structure of a clinical catheter, which could be considered as a pipe with small branches, naturally supports propagation of UGWs^27^. Resulting from the wave reflections and mode conversions at the boundaries of a solid pipe, UGWs exhibit distinctive characteristics such as dispersion and axis-symmetric/axis-asymmetric behaviours. Here a new anti-biofilm catheter prototype was proposed, named as UGW-ET. By combining an ET with a group of UGW transducers, UGWs were excited and propagated along the ETs, stopping bacteria aggregation on ETs’ surfaces. The general protocol was based on theoretical analysis of UGW modes, mode/frequency selection, calibration of UGW vibration, as well as wave coupling techniques. *In vitro* experiments targeting *P*. *aeruginosa* (ATCC 27853) were carried out, biofilm prevention capability of UGW-ET was evidenced through both bacteria counting and microscopic observations.

## Results

### UGW-ET prototype

**Figure 1:**
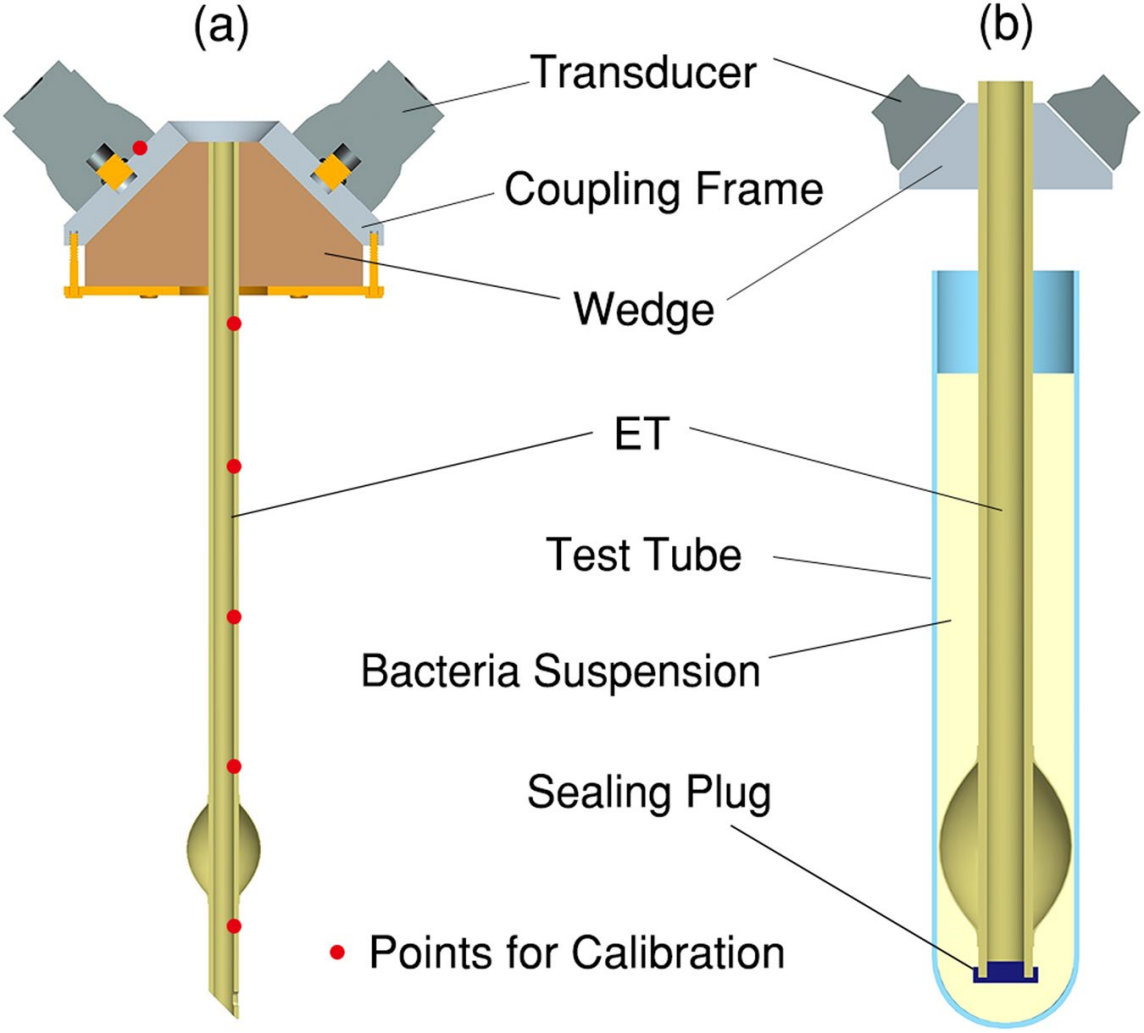
(**a**) Schematic illustration of UGW-ET; (**b**) *in vitro* configuration of UGW-ET.

The design of the catheter prototype is illustrated in Fig. 1a. A 26-cm ET (ET-02S, Bever, Hangzhou, China), whose outer and inner diameters were respectively 10.4 mm and 7.0 mm, was used as the main body. The upper end of the ET was coaxially inserted into a circular frame made of aluminium. Four piezoelectric ceramic transducers (PZT), electronically connected in parallel, were equally-spaced in circumferential direction around the frame. The piston transducers, with their nominal frequencies of 50 kHz and diameters of 38 mm, were carefully calibrated to guarantee nearly identical performances. A wedge made of acrylonitrile butadiene styrene (ABS) was used as the base of the transducer group. Hence, UGWs were guided into the tube at an angle of 45° rather than perpendicularly. The reason to choose 45° was, most UGW modes consisted of vibration components in both axial and radial directions, while 45° incidence offered equal opportunities to excite vibrations in either direction^28^. Medical ultrasound coupling agent (AQUASONIC^®^ 100, Parker, Fairfield, NJ, USA) was used to facilitate wave transmission through all interfaces, helping to minimize wave reflections. For *in vitro* experiments, the free end of the ET was sealed before dipped into a 50-mL Falcon test tube, as is depicted in Fig. 1b.

### UGWs Propagation without wedge and coupling

To detect the radial vibrations on ET surfaces, a laser vibrometre based on heterodyne interference was used. Measurements were performed at six spatial points, locating at 0 (on transducer surface), 1, 5, 10, 15, and 20 cm away from the wedge, as marked out by red dots in Fig. 1a. Ten replicated measurements were performed at each location, while 100 continued pulse sequences were acquired and averaged to reduce the signal-to-noise ratio (SNR) in each individual measurement.

**Figure 2:**
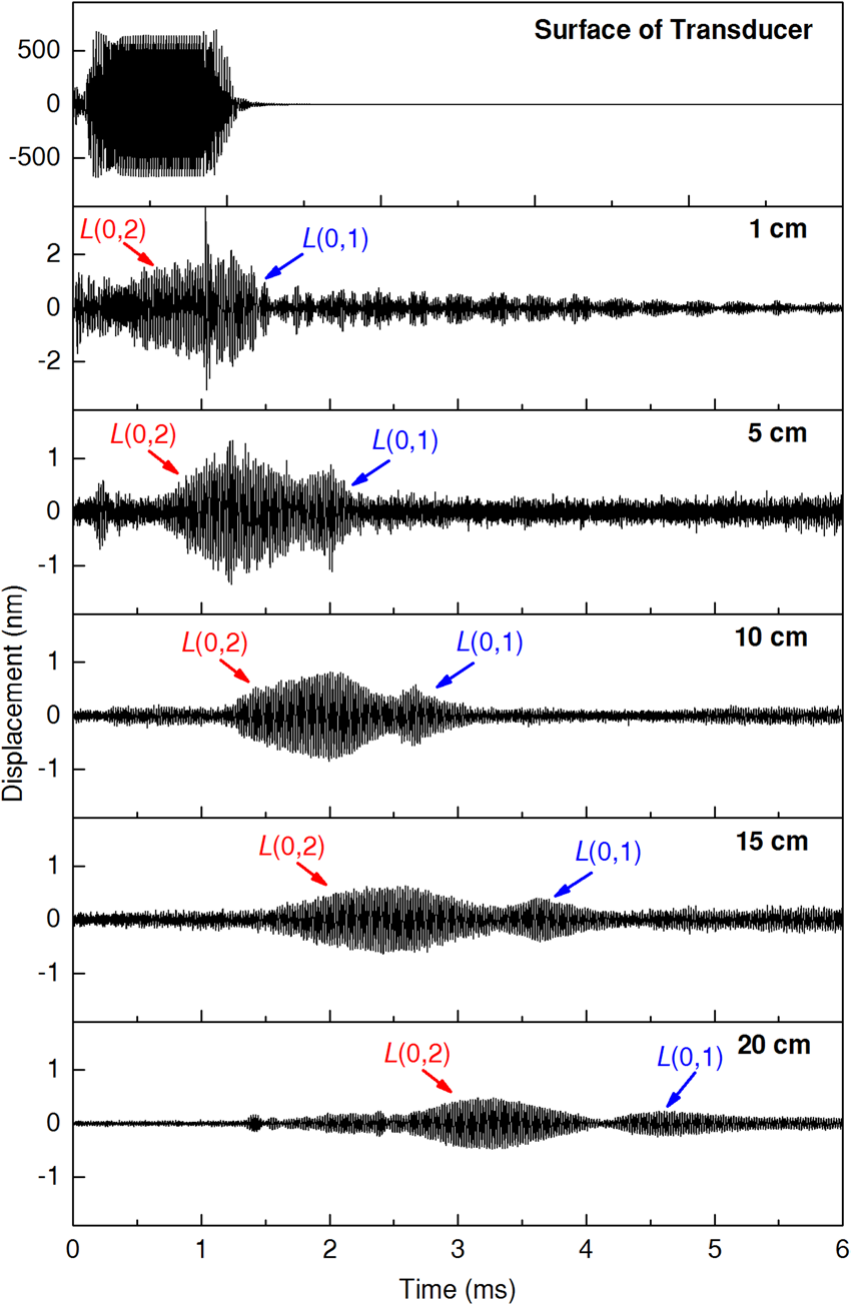
Representative UGW displacements detected at the pre-selected locations. Incidence was applied perpendicularly to ET surface, no coupling medium was used, driving frequency was 50 kHz.

For demonstration purpose, at first the 45° wedge and coupling medium were not applied, thus only normal incidence of the transducer was adopted to an ET. Representative motion displacements detected at the pre-selected positions are plotted in Fig. 2. It is observed that UGWs were successfully generated along the ET, and their dispersive behaviour could be identified easily. As was expected, two longitudinal modes, *L*(0, 1) and *L*(0, 2), played the major roles in the recorded waveforms, with their propagation velocities differed from each other. Both wave packages tended to expand themselves when going away from the source, suggesting the UGW modes were still dispersive due to the limited bandwidth of the driving signal. Several small packages also existed, indicating more modes were actually present, although possessing very low energy.

**Figure 3:**
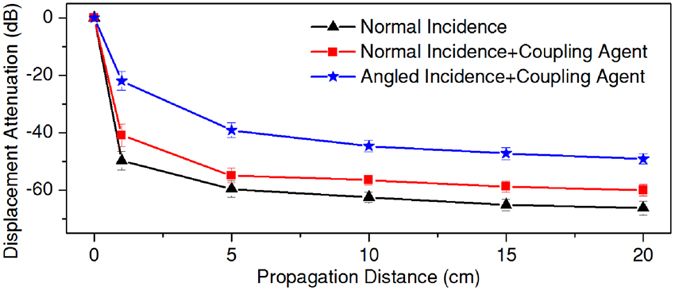
Attenuation of UGW displacement amplitudes as functions of propagation distance. Driving frequency was 50 kHz.

Another concern that arises from Fig. 2 is that, UGW wave packages not only expanded, but also decayed quickly when travelling. In fact, significant attenuation could impair wave transmission, playing a negative impact in device performance. To make a quantitative assessment, Fast Fourier Transform (FFT) was conducted on all recorded waveforms, from which the amplitudes of 50-kHz components were extracted. UGW attenuation as a function of propagation distance was then calculated and illustrated in Fig. 3 (black line). The results show that, vibration amplitudes decreased rapidly as UGWs propagated ahead, especially within the first 5-cm area. This propagation loss could be contributed by: a) air gaps at the interfaces; b) wave reflections on boundaries; and c) damping of the ET material.

### Improvements from wedge and coupling

Vibration amplitudes should be crucial for biofilm prevention, hence minimized UGW propagation loss was expected. By adopting the 45° wedge and coupling medium, comparisons were made among three excitation strategies, *viz*., normal incidence (excitation strategy A), normal incidence with coupling gel (excitation strategy B), and 45° incidence with coupling gel (excitation strategy C). As is shown in Fig. 3, application of coupling gel increased the wave amplitudes by 6–10 decibels. By combining the angled wedge and coupling gel, wave amplitudes were lifted by 15–25 decibels, exceeding 5 nm at the end of the ET (20 cm). Therefore, excitation strategy C should be considerably helpful in facilitating UGW transmission.

### *In vitro* results of reduced bacteria aggregation

Five groups of experiments were carried out at different UGW amplitudes. For each group, vibration amplitude was measured at 1 cm from the wedge, and the second plot in Fig. 2 represents the analogic waveform measured under excitation strategy A. The amplitudes of the 50-kHz components, denoted as *D*_50-1_, were then extracted from the recorded waveforms via FFT algorithm, and used as the indicator of each group. For individual groups (from group 1 to 5), *D*_50-1_ was 0 (sham group), 0.05 nm, 0.5 nm, 5.0 nm and 50 nm, respectively.

**Figure 4:**
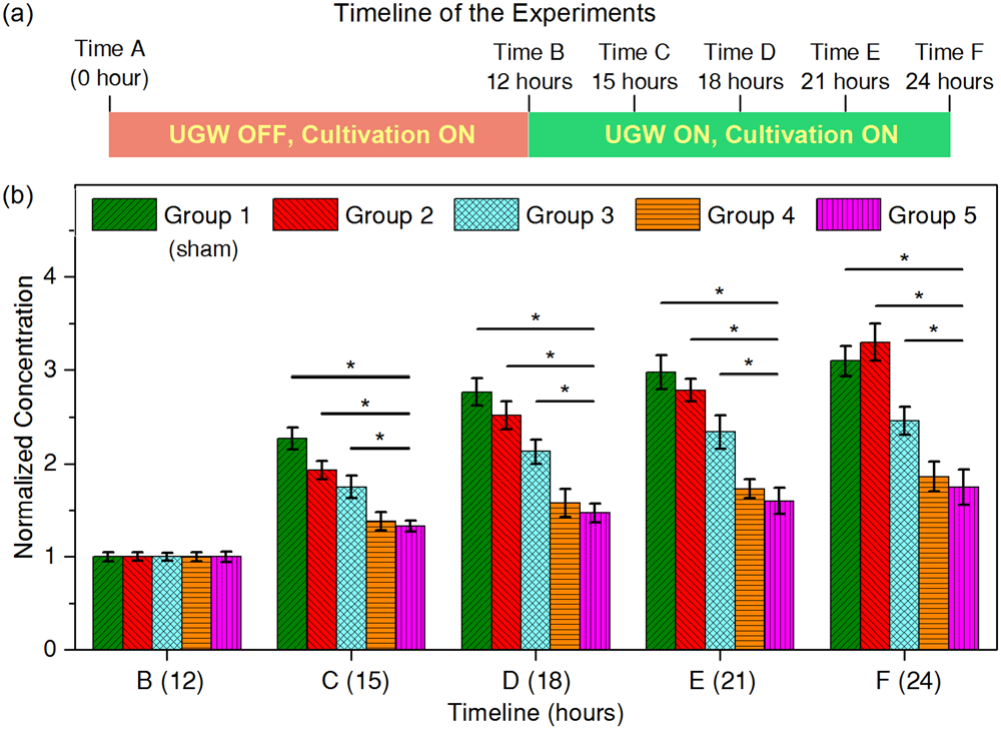
(**a**) Timeline of the experiments. (**b**) Temporal variations of normalized bacteria concentrations for Groups 1–5, corresponding to *D*_50-1_ = 0 (sham group), 0.05 nm, 0.5 nm, 5 nm, and 50 nm, respectively. UGWs were turned on for Groups 2–5 between Time B (12 hours) and Time F (24 hours). Wedges (45°) and coupling gel were used, UGW frequency was 50 kHz. *D*_50-1_ was the FFT-extracted 50 kHz amplitude detected at 1 cm from the wedge. Significant differences can be identified between groups with a p-value less than 0.05 (^*^ *p* < 0.05).

A timeline was setup for a 24-hour experimental period, as is described in Fig. 4a. For each group, sixteen testing tubes were kept for *P. aeruginosa* (ATCC 27853) cultivation from time A (0 hour) to time B (12 hours). Then, three tubes were taken out from each group for bacteria counting. In addition, one tube out of the three was also used for microscopic check. UGW excitation was turned on immediately after time B for all groups except Group 1. Then, three samples were taken out from each group for bacteria counting at time C (15 hours), D (18 hours) and E (21 hours), respectively, with UGW kept working. At time F (24 hours), the last four tubes in each group were taken out for microscopic examination, after which three of them were used for bacteria counting. To achieve bacteria counting, *P. aeruginosa* aggregated on each ET were dispersed into 2 mL phosphate buffer saline (PBS), and the colony concentration was measured.

Results in Fig. 4 show that, the number of aggregated bacteria increased as the timeline progressed ahead in all groups, but the growth rate tended to decrease especially after time D (18 hours). With the application of UGWs, aggregation of bacteria on ET surfaces was significantly inhibited, informing the dramatic reduction of biofilms. The statistical analysis results suggested significantly better biofilm suppression effect could be observed with enhanced UGW amplitude. However, elevating the vibration level to higher than 5 nm would not be necessary, because no statistically significant difference was observed between the last two groups, suggesting the anti-bacterial effect tended to saturate as *D*_50-1_ > 5 nm.

### Microscopic proof of biofilm prevention

**Figure 5:**
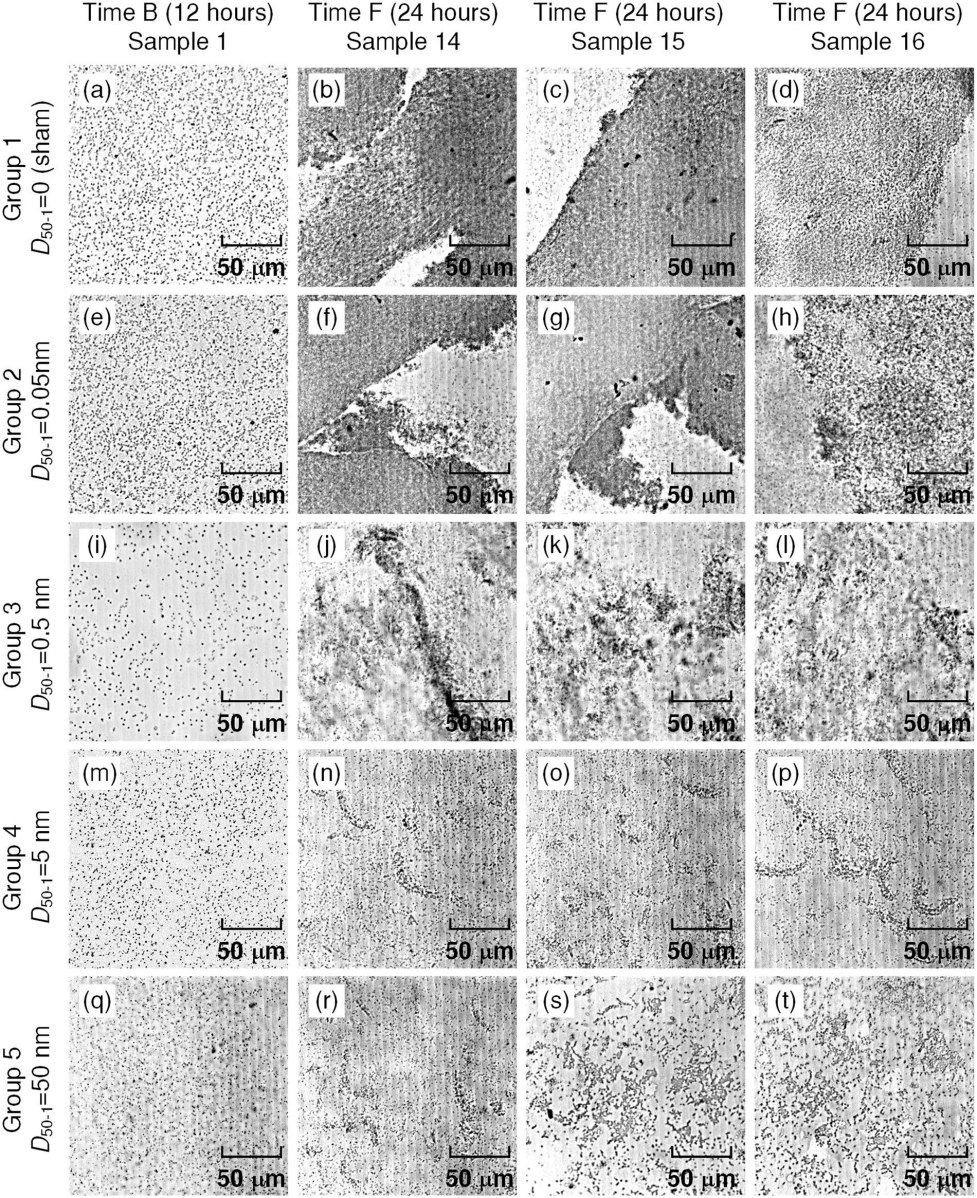
Microscopic images of biofilms for Groups 1–5, corresponding to *D*_50-1_ = 0 (sham group), 0.05 nm, 0.5 nm, 5 nm, and 50 nm, respectively. UGWs were turned on for Groups 2–5 between Time B (12 hours) and Time F (24 hours). Wedges (45°) and coupling gel were used, UGW frequency was 50 kHz. *D*_50-1_ was the FFT-extracted 50 kHz amplitude detected at 1 cm from the wedge.

Figure 5 gives the microscopic pictures taken at time B (12 hours, one sample for each group) and time F (24 hours, three samples for each group). In the first column, *P. aeruginosa* bacteria are sparsely-distributed on ET surfaces for each group, hence biofilms had not formed at time B. When the timeline approached 24 hours (time F), the three samples of Group 1 (sham group) showed massive biofilm adherence on ET surfaces. By activating UGWs propagation, the situation was totally changed. For *D*_50-1_ = 0.05 nm (Group 2), although biofilms also deposited on ETs, they appeared less dense compared to Group 1. With *D*_50-1_ raised to 0.5 nm, bacteria aggregation only emerged at several localized areas (Group 3). By further increasing the vibration amplitude to 5 nm (Group 4), although the numbers of bacteria were higher than those of Group 1, biofilms were completely absent.

In the microscopic results of both Group 4 and Group 5, biofilm formations were inhibited effectively by UGW propagation. Comparison between these two groups again showed that, increasing UGW vibration to an excessively high level would not be much beneficial, as is indicated by Fig. 4.

## Discussion

A substantial question underlying UGW-ET is why UGW was chosen for biofilm prevention. In fact, UGW propagation is not optional but inevitable for ultrasound-mediated catheters, as guided modes always exist inside tubes following the nature of wave motion^28, 29^. Therefore, to achieve high-performance ultrasonic catheters, effective regulation of UGW vibration is necessary and important.

Although previous studies have speculated an interaction between biofilm formation and ultrasonic vibration, the inherent mechanism is poorly understood^30^. Figures 4 and 5 indicate that, stopping bacteria from depositing is the key in UGW-assisted biofilm prevention. It is therefore unsurprising that UGW-ET achieved better performance when the vibration amplitude became higher. However, it could be a little confusing that different vibration amplitudes work for different studies, i.e., the current work used 0.05–50 nm, while those in previous studies spanned a wider range from 0.2 nm to 800 nm^9, 10^. Apart from other factors like frequency and configuration, it is essential that vibrations be introduced into catheters with high efficiency, a duty of physicists rather than bacteriologists. The examples in Fig. 3 show that, 45° incidence combined with effective coupling did an excellent job in facilitating UGW generation. It should also be noted that, ultrasonic vibrations exceeding specified thresholds might also cause thermal effects in human tissues. If ultrasonic catheters work at relatively high amplitudes^31^, potential damage could be imposed on patients.

**Figure 6:**
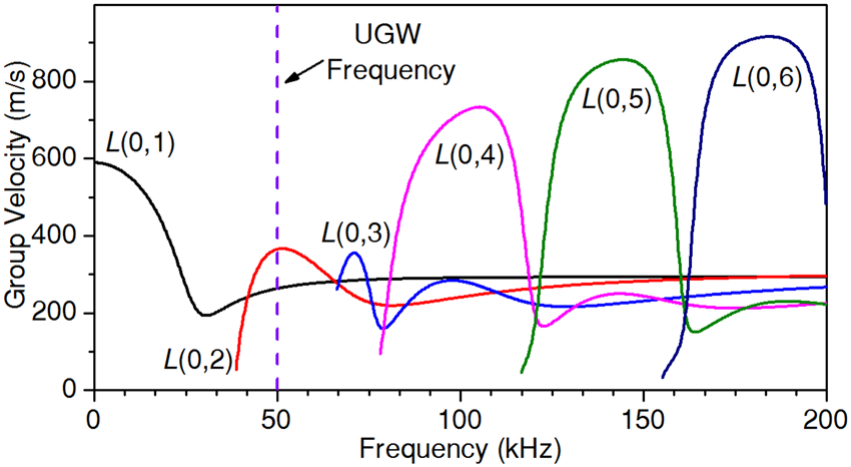
Group velocity dispersion curves of longitudinal UGW modes along the ET.

Selection of working frequency is also critical, during which the inherent dispersive characteristics should be carefully examined. As is indicated in Fig. 6, both *L*(0,1) and *L*(0,2) modes should be weakly dispersive at 50 kHz. However, in Fig. 2 wave package expansions still emerged, though slightly. If UGWs worked at a strongly dispersive frequency, wave amplitudes could had decreased more harmfully due to wave expansion^32, 33^. For frequency selection, one should also keep away from the 20-kHz audible limit, since the noise from that could be clinically unacceptable.

An even more interesting question is how to quantify the impact of UGW on the bacteria reproducing, which has been observed with 70 kHz ultrasound for *P. aeruginosa* and 95–220 kHz for *S. epidermidis* ^13, 23^. It is expected that, gene expression reprogramming and protein synthesis could be influenced, and cell-cell communications between microorganisms might be disrupted^11, 22^. Also, it could be very intriguing if UGW-ET could be combined with other techniques, such as antibiotics. Interruption of biofilm generation caused by UGWs could lead to the destruction of the protective growth mode of bacteria^9, 10, 12^, therefore antibiotics should work at higher efficiency. Furthermore, combining UGW-ET with catheter-coating based biological/chemical technique is also promising and feasible. Since the prevalent anti-bacterial coatings are generally of nano/micrometre size^17–20^, their influence on physical parameters of catheter material and propagation characteristics of UGWs could be negligible. Therefore, changing the surface properties may lead to an adaptive response of the bacteria to attach in a different manner or thrive via the expression of stress responses, which may in turn affect their response to the UGW treatment.

The UGW-ET prototype is clinically applicable and compatible. Using UGWs rather than other wave types brings the advantage of long-range wave transmission. Since targeted sonication is unnecessary as is described in Fig. 1b, it is only required that part of a catheter remains outside the patient’s body for UGW generation. This device also works for bent catheters. Bending pipes also support the transmission of UGWs, while mode dispersion and motion amplitude could be slightly influenced. For example, bending-caused transmission loss of *L*(0,2) mode would be less than 20%, which is valid for different pipe thicknesses and bend radii^34^. It should also be noted that, the coupling gel applied in UGW-ET is originally designed for medical use. According to the product manual, it is completely aqueous, bacteriostatic, non-sensitizing and non-irritating. This viscous gel medium has no harmful components such as formaldehydes or spermicides, it is sterilized and should be safe for clinical patients.

Future efforts are needed to achieve UGW-ETs of better performance. For example, it is of great interest to check how axial and radial vibration components play their roles, and how acoustic emission parameters (e.g., frequency, waveform, pulse repetition frequency, pulse length, etc.) could be optimized. Other types of catheters could also be modified with the current UGW-based technique.

## Conclusion

One key factor to stop biofilms on catheters is to prevent bacteria from depositing on catheter surfaces. By exploiting the vibrational nature of ultrasound, a UGW-ET prototype has been proposed and proven successful in preventing *P. aeruginosa* biofilms. It is concluded that, UGW vibration could be better employed when its inherent characteristics (*e.g*., dispersion and attenuation) are considered. Mode analysis and frequency selection are hence necessary and important, such that catheter vibration could be predictable and controllable. Also, an optimized performance of UGW-ET could not be realized unless UGW transmission loss could be minimized. To resist UGW attenuation, wave coupling technique should be applied properly, *e.g*., 45° incidence and coupling medium used in this work. Finally, quantitative relations between anti-biofilm capability and UGW vibrations should be established, during which accurate motion calibration are needed. For preventing *P. aeruginosa* biofilms on a soft polyvinyl chloride (PVC) ET, UGW vibration amplitudes ranged from 0.05–50 nm are suggested. This work is helpful in understanding the physical mechanism of ultrasound-mediated anti-biofilm effect, and will inspire the development of new indwelling tubes of anti-bacterial capability.

## Methods

### Material parameters of UGW-ET

According to the product manual, the ETs were made of soft PVC whose material parameters are: Young’s modulus *E* = 6 × 10^6^ Pa, Poisson’s ratio *σ* = 0.47 and density *ρ* = 1290 kg/m^3^. Lamé constants of soft PVC were calculated as: *λ* = *Eσ*/[(1 + *σ*)(1 − 2*σ*)] and *μ* = *E*/[2(1 + *σ*)]. The coupling frame was made of aluminium, whose parameters are: density *ρ*_AL_ = 2700 kg/m^3^, longitudinal sound velocity *c*_L,Al_ = 6226 m/s, and shear sound velocity *c*_T,Al_ = 6226 m/s.

### Dispersive nature of UGWs

The ET was modelled as a circular pipe that supported three different types of UGW modes, *i.e*., longitudinal modes *L*(0,*m*), torsional modes *T*(0,*m*), and flexural modes *F*(*n*,*m*), with *n* and *m* being positive integers. *L* and *T* modes were axis-symmetric while *F* modes were asymmetric. Except for *T*(0,1), all other modes exhibited velocity-dispersive characteristics which were related to the wave number *k*, the pipe thickness *a*, as well as the longitudinal and shear wave velocities ${c}_{L}=\sqrt{(\lambda +2\mu )/\rho }$ and ${c}_{T}=\sqrt{\mu /\rho }$.

Two types of mode inhibition techniques were adopted to reduce the complexity of wave propagation. First, by selecting an axis-symmetrical excitation configuration, *F* modes could be totally eliminated. However, due to the limitation of transducer fabrication, an ideal axis-symmetrical excitation was hard to achieve. The solution was to use a number of identical transducers, which were located at the same axial coordinate of the ET, and were equally spaced in the circumferential direction^29^. Second, by confining the excitation of each transducer to the axial-radial plane, *T* modes also disappeared in the wave packages.

Then, propagating along the pipe were mainly *L* modes. A number of authors have reported the use of Lamb waves for inspection of pipes and tubes^35, 36^. When a pipe and a plate had the same thickness, the dispersive relation of *L*(0,1) and *L*(0,2) UGW modes respectively resembled those of *A*_0_ and *S*_0_ Lamb modes, while other UGW modes also had their Lamb analogies^37^. The resemblance between UGW modes and Lamb modes could be concluded by comparing Fig. 6 in this work with Fig. 2b in ref. 37. It is particularly important that, around the weakly dispersive frequency (50 kHz in Fig. 6, 3200 kHz in ref. 37), the dispersive characteristics between UGW modes and Lamb modes were almost the same. Therefore, in this work dispersion relations of Lamb waves were used for UGW analysis,

1 $$\frac{\tan (qa)}{\tan (pa)}=-\frac{4{k}^{2}pq}{{({k}^{2}-{q}^{2})}^{2}}$$

Here $p={\omega }^{2}/{{c}_{L}}^{2}-{k}^{2}$, $q={\omega }^{2}/{{c}_{T}}^{2}-{k}^{2}$, and *a* was the pipe thickness. The group velocity (the speed at which UGWs propagated along the ET) dispersion curves based on Eq. (1) are presented in Fig. 6. Each curve tells how an individual mode travelled at different speeds when changing the working frequency.

### Frequency selection

According to Fig. 6, there existed six modes within the frequency range 0–200 kHz, *i.e*., *L*(0,*m*), *m* = 1~6. Each mode had a specified cut-off frequency. The excitation frequency was selected by following two criteria: a) there existed less modes at the selected frequency, as too many modes would make data analysis much complicated; and b) UGW modes were weak dispersive at the selected frequency, since strong dispersion could lead to severe waveform distortion^38^. Based upon these, the working frequency was chosen as 50 kHz, at which *L*(0,1) and *L*(0,2) modes were capable of propagating, both exhibiting weak dispersion.

### UGW excitation

An arbitrary waveform generator was used to produce tone-burst signals of 50-kHz central frequency, 10% duty cycle, 100 Hz pulse repetition frequency (PRF), and up to 200 mV peak-to-peak amplitudes (V_p-p_). After being amplified with an RF power amplifier of 53-dB gain, the signals were used to drive the electrically-connected transducer group.

### Bacteria cultivation

A strain of *P. aeruginosa* ATCC 27853 was purchased from American Type Culture Collection (ATCC, USA). Bacteria suspension was then obtained by overnight culturing of *P. aeruginosa* at 37 °C with 5% CO_2_ and 150 rpm shaking. The culturing medium was prepared beforehand by dissolving 15 g powdered Tryptone soya broth (TSB; Oxiod, Hamshire, Britain) in 500 mL distilled water. To prepare testing samples, 4 mL of the culturing medium was added into each test tube, and sterilized through high pressure steam at 121 °C for 15 minutes. Before UGW-ET was inserted into each tube, 10 μL bacterial suspension was added into it. Finally, all samples were put in a bacteria incubator (37 °C, 5% CO_2_) for experiments.

### Bacteria counting and microscopic inspection

After samples were taken out from the incubator, one-time cleaning was gently performed for each test tube, during which 2-mL PBS was applied by pipetting. Bacteria counting was achieved through the following procedure: a) vibrating biofilm off from each ET along its 20-cm length using a cell crusher, which worked at 20 kHz and 3-W output for two 30-second pulses; b) blending the bacteria from each sample into 2 mL PBS; and c) calculating bacterial colony concentration through turbidimetry, which was based on optical density measurements at a wavelength of 600 nm (OD600). The concentration values measured at time B (12 hours) were averaged for each group, and used as normalization standards for their following data. It should be noted that, vibration amplitude of the cell crusher probe should have been several orders higher than that of the UGW transducers. As a result, vibration on the ET surface induced by the probe should also be more intense than that caused by UGWs.

For microscopic observations, each sample was examined using an inverted microscope with 40X lens working in bright field mode, immediately after the PBS cleaning step.

### Statistical analysis

One-way analysis of variance (ANOVA) was used to compare data among groups using Origin Software (OriginLab Co. Northampton, MA, USA). A p-value less than 0.05 was considered a statistically significant difference. Data are presented as means ± standard deviation (s.d.).
